# 细胞治疗与造血干细胞移植新模式的探索与实践

**DOI:** 10.3760/cma.j.cn121090-20230928-00150

**Published:** 2024-02

**Authors:** 曦 张, 瑞昊 黄

**Affiliations:** 1 陆军军医大学新桥医院血液病医学中心，创伤、烧伤与复合伤国家重点实验室，全军血液病中心，重庆市医学重点学科，重庆 400037 Medical Center of Hematology, Xinqiao Hospital, State Key Laboratory of Trauma and Chemical Poisoning, Army Medical University, Chongqing 400037, China; 2 金凤实验室，重庆 400037 Jinfeng Laboratory, Chongqing 400037, China

**Keywords:** 血液肿瘤, 细胞治疗, 造血干细胞移植, Hematological malignancies, Cell therapy, Hematopoietic stem cell transplantation

## Abstract

造血干细胞移植为多种血液学疾病尤其是恶性血液病提供了有效治愈办法，其治疗体系不断优化，供者来源不断扩大，适应证不断拓展，疗效也在一定程度上取得了突破。目前造血干细胞移植技术在大部分血液病中的地位仍不可撼动，但是原发病复发及造血干细胞移植相关并发症仍是影响患者长期生存及生活质量的两大临床困境。以嵌合抗原受体T（CAR-T）细胞治疗为代表的细胞治疗在难治/复发B细胞恶性肿瘤的治疗上取得了突破性进展。细胞治疗相比传统药物具有独特的体内代谢动力学特点，依靠免疫特异性识别及干细胞的修复能力，目前在血液肿瘤治疗及移植并发症处理中崭露头角，目前多项临床研究已经初步展现了细胞治疗与造血干细胞移植结合的新诊疗模式。

## Abstract (en)

Hematopoietic stem cell transplantation provides an effective cure for various hematological diseases, especially malignant hematological diseases, its treatment system has been continuously optimized, the source of donors has been expanding, the indications have been expanding, and the therapeutic effect has also made breakthroughs to a certain extent. At present, the status of hematopoietic stem cell transplantation technology in most hematological diseases is still unshakable, but the recurrence of the primary disease and complications related to hematopoietic stem cell transplantation are still two major clinical challenges that affect the long-term survival and quality of life of patients. Cell therapy represented by chimeric antigen receptor T (CAR-T) has made breakthrough progress in the treatment of refractory/recurrent B-cell malignancies. Compared with traditional drugs, cell therapy has unique in vivo metabolic characteristics, relying on immune specific recognition and the repair ability of stem cells. It is currently emerging in the treatment of blood tumors and the management of transplant complications. Multiple clinical studies have preliminarily demonstrated a new diagnostic and therapeutic model combining cell therapy with hematopoietic stem cell transplantation.

细胞治疗于2011年被全国科学技术名词审定委员会定义为“利用患者自体（或异体）的细胞对组织、器官进行修复的治疗方法”。在血液病治疗中，目前最常见的细胞治疗形式就是造血干细胞移植（HSCT），是目前大部分恶性血液病唯一的治愈手段。HSCT可主要通过移植前预处理清髓化疗及移植物抗白血病（GVL）效应达到清除白血病细胞的目的，后续也衍生出供者淋巴细胞输注（DLI）、微移植等治疗方式。但传统HSCT及相关治疗目前仍有一定局限性。首先，基于大剂量化疗/全身放射的预处理方案，肿瘤杀伤能力受安全性影响有一定上限，尤其是对于白血病干细胞杀伤能力较弱，这也是后期导致患者复发主要原因。其次，传统免疫抑制剂在预防或治疗移植物抗宿主病（GVHD）的同时会抑制GVL效应，单纯通过免疫抑制药物管理GVHD与复发及感染难以达到理想平衡。此外，移植后植入不良及移植血管内皮损伤相关并发症目前没有好的有效手段。

CAR-T细胞疗法将细胞治疗引入了新时代，基因编辑技术的蓬勃发展也赋予了细胞治疗更强的抗肿瘤作用。在血液系统疾病的治疗中，以CAR-T细胞治疗为首的系列免疫细胞治疗在诱导缓解方面可独当一面，甚至在某些特定条件情况下挽救HSCT治疗无效的患者。但是，随着确证性临床试验的深入，免疫细胞治疗仍存在高复发、脱靶等问题需要进一步治疗。除免疫细胞治疗外，随着细胞鉴定及分选技术的提升，以间充质干细胞（MSC）为代表的干细胞疗法可不受人类白细胞抗原（HLA）限制用于多种自身免疫性疾病，对于植入不良、GVHD等并发症也有治疗、预防作用。本文就细胞治疗与HSCT新模式探索与实践进行评述。

一、细胞免疫治疗和HSCT的结合

传统的自体造血干细胞移植（auto-HSCT）主要通过大剂量化疗达到杀伤肿瘤细胞的目的，自体干细胞回输后不会产生新的抗肿瘤效应。早期的过继性细胞疗法通过体外肿瘤细胞共培养及细胞因子刺激等方式提高T细胞或自然杀伤（NK）细胞的抗肿瘤效应，但总体疗效难以达到临床需要的治疗作用。CAR-T细胞治疗的出现改变了这一局面，通过抗原抗体特异性识别的方式，CAR-T细胞抗肿瘤效力得到极大提升。CAR-T细胞疗法上市以来，免疫细胞治疗目前已经成为难治、复发B细胞血液恶性肿瘤治疗中的生力军[1]。目前国内已获批用于治疗难治、复发B细胞非霍奇金淋巴瘤（B-NHL）、急性B淋巴细胞白血病（B-ALL）及多发性骨髓瘤（MM）[2]。但是细胞免疫治疗还有一些尚未被解决的问题，复发仍然是需要解决的首要问题，因此和HSCT“强强联合”应该是解决难治复发血液肿瘤比较好的策略。

首先在HSCT前，对于B-ALL，CD19 CAR-T细胞疗法单用虽然缓解率高，但2年复发率较高，难以取得长期生存获益，目前多项国内外研究均表明CAR-T诱导难治/复发B-ALL缓解后桥接异基因造血干细胞移植（allo-HSCT）能显著提高患者的无白血病生存（PFS）[3]。而对于allo-HSCT而言，移植前无白血病状态对预后至关重要，所以目前国内外对CAR-T细胞治疗达到完全缓解的患者桥接移植能获得显著生存获益基本达成了共识。除外目前已上市的CAR-T细胞治疗外，针对急性髓系白血病（AML）及T细胞恶性血液肿瘤的免疫细胞治疗在初步探索中取得一定疗效，但也遇到了一些问题。对于AML，由于其肿瘤微环境的特异性CAR-T细胞疗效有限，且靶向AML的免疫细胞疗法难免会导致造血干细胞损伤，长时间的重度骨髓抑制使治疗相关风险显著提高。allo-HSCT可清除剩余CAR-T细胞并重建免疫及造血[4]。此外，通过基因编辑技术使患者自身健康细胞的靶点无法被CAR-T细胞识别是防止CAR-T细胞脱靶毒性的可行途径，已有研究表明使造血干细胞膜表面的CD33错误表达可防止靶向CD33的CAR-T对造血干细胞进行杀伤，达到“分辨敌我”的目的，相当于auto-HSCT的变式与CAR-T细胞联合，目前该研究已在临床前阶段，这种形式也可能是未来降低CAR-T细胞脱靶效应的有效手段。

NK细胞被证实有抗肿瘤作用，安全性高，但抗肿瘤效应相对较弱。早期也有运用NK细胞强化移植后免疫缓解GVHD的相关报道[5]。目前NK细胞疗法在AML中的安全性及有效性在几项探索性临床研究中已得到初步验证，但NK细胞续存性较差，目前桥接HSCT是延长无复发生存的有效手段[6]。对于T细胞血液肿瘤，目前已发表的探索性临床研究表明T-ALL/T-LBL患者自体T细胞制备的CAR-T细胞没有成瘤风险。但是，靶向T细胞肿瘤相关抗原（TAA）的CAR-T细胞有导致治疗相关免疫缺陷风险，而allo-HSCT是有效解决途径之一[7]。CD19 CAR-T细胞正挑战auto-HSCT在B细胞NHL中的二线治疗地位，一项研究表明对于一线治疗无效的NHL患者CD19 CAR-T细胞显著优于auto-HSCT，同期另一项“背靠背”研究则表明两者疗效无统计学差异。虽然针对这一问题目前尚无定论，但已有研究表明CAR-T细胞后桥接auto-HSCT有克服TP53突变、高级别淋巴瘤患者的不良预后的潜力[8]。总的来说，CAR-T治疗可在移植前达到高度缓解，为移植提供机会，而移植可及时解除CAR-T细胞治疗的不良反应并巩固疗效。

在移植后，复发仍是移植后患者死亡的首要原因，DLI可在一定程度上提升GVL效应，但是对于确证进展的难治、复发急性白血病，DLI疗效尚无法满足临床需求且会提高GVHD的风险。对于移植后复发的B-ALL，供者来源的CAR-T细胞疗法作为挽救治疗可再次使本病达到完全缓解或抢先清除微小残留病（MRD），且不会显著提升GVHD风险，在一定程度上实现了GVHD与GVL分离[9]–[10]。除抗肿瘤作用外，免疫细胞疗法在移植后抗病毒感染方面也有一定成效，病毒特异性T细胞（VST）细胞输注可有效降低HSCT后病毒感染导致的相关并发症发生率，是除抗病毒药物以外的有效补充，为移植后患者保驾护航[11]–[12]。

二、其他类型干细胞和HSCT的结合

针对allo-HSCT尤其是单倍体造血干细胞移植（haplo-HSCT）并发症管理的主要矛盾在于免疫抑制剂的运用面临GVHD与GVL失衡问题。常规免疫抑制剂在抑制GVHD的同时会导致复发和感染风险的升高，由于患者情况不同，免疫抑制剂在预防和治疗时剂量不易把控，GVHD与GVL失衡问题难以解决。目前已有多项临床研究证明，间充质干细胞（MSC）在降低并治疗GVHD的同时，可保留GVL效应，且没有明显的不良反应[13]–[14]。其中代表的MSC目前已获批急性GVHD二线治疗。我中心率先将MSC用于慢性GVHD的预防，证实了在移植后100 d输注MSC对于慢性GVHD，尤其是预后不良的肺慢性GVHD有很好的预防作用，最近研究也显示移植后早期（移植后45 d）输注MSC能够有效降低重度慢性GVHD发生率且对复发没有显著影响，显著提升GVHD无复发生存（GRFS）；然而，多项Meta分析提示MSC治疗效果并不稳定，且MSC来源、输注量、输注时间对疗效的影响较大，这与MSC异质性有关[15]，这也是目前干细胞治疗的主要争议所在。由于干细胞的多能性，其作用机制相对复杂，结果一致性相比传统药物较弱，但是目前的临床研究结论还是倾向于MSC有助于降低GVHD发生率且不会增加复发风险。针对这一问题，南方医院刘启发教授团队联合单抗类药物实现了MSC的增效。除预防、治疗GVHD外，鉴于MSC在正常的造血微环境中起到重要支持作用[16]，目前研究已证明MSC与造血干细胞共同输注可促进造血重建，协助血小板恢复[17]–[18]。

此外，内皮祖细胞（EPC）是内皮细胞的前体细胞，也是造血微环境的重要组成部分，在造血干细胞植入不良患者中，骨髓EPC数量和功能严重损伤，是植入不良的独立危险因素，修复EPC可以促进移植后造血重建，降低植入不良等移植并发症；肝窦阻塞综合征/肝小静脉闭塞症（SOS/VOD）也是致死率较高的早期并发症，其发病机制主要与内皮损伤相关，EPC可有效修复内皮从而减低SOS/VOD的发生，临床研究正在开展之中[14]。

三、展望

免疫细胞具有高效抗肿瘤效应，MSC或EPC治疗具有调节免疫、抗炎及修复的效应且毒副作用较轻，为恶性血液肿瘤移植后降低复发及相关并发症的问题提供了有效解决途径。但是目前两种疗法的结合仍有一些争议，譬如如何保证移植后CAR-T细胞的续存能力？进一步对CAR-T治疗后的患者分层，高危患者尽早进入移植流程，低危患者则可以继续观察，这仍需要进一步完善。针对MSC的异质性，如何进一步明确治疗优势细胞亚群或配伍药物也需要进一步的探索。展望未来，免疫细胞治疗的发展仍需要依靠基因编辑技术及免疫耐受机制的探索，目前在临床的运用还需要根据病情严重程度和HSCT等疗法相结合。而MSC等干细胞疗法的争议主要在于，作用机制复杂难以通过单一途径解释，目前单独干细胞疗法难以达到全面预防和治疗并发症的目的，多种干细胞联合可能获得更全面的防治作用。得益于上述细胞产品的研发，基于基因编辑技术的干细胞定向编辑可能开启auto-HSCT治疗单基因遗传病的时代。总的来说，细胞治疗可进一步提升HSCT体系的治疗效果并降低相关并发症，但精准有效联合手段的选择仍需要进一步探索。
